# Regional differences in the distribution of melanocyte-containing hair bulbs in the skin of male albino rats

**DOI:** 10.1371/journal.pone.0336110

**Published:** 2025-11-05

**Authors:** Nayuki Numata, Aisa Ozawa, Motoharu Sakaue

**Affiliations:** Laboratory of Anatomy II, Department of Veterinary Medicine, School of Veterinary Medicine, Azabu University, Sagamihara, Kanagawa, Japan; Wuhu Hospital Affiliated to East China Normal University, CHINA

## Abstract

Hair gets its color from melanin produced by melanocytes in the hair matrix. The coloration patterns observed in most terrestrial mammals arise from the diverse color combinations within their fur, which depends on the distribution pattern of melanocyte-containing hair follicles. Albino rats genetically produce no melanin and their coats are thus white, but we speculated that melanocytes differentiate and localize within these rats’ hair matrix. We conducted a reverse transcription-quantitative polymerase chain reaction (RT-qPCR) analysis, which revealed both the mRNA expressions of two melanocyte markers (dopachrome tautomerase and tyrosinase) in skin of male albino (SD, Wistar, and F344) rats and the differences in the markers’ expression levels among skin areas. Immunohistochemistry using anti-Dct antibody demonstrated that immunopositive cells, i.e., melanocytes, were localized in the rats’ hair matrix, and that melanocytes containing hair bulbs were distributed in head, dorsal thorax, and dorsal midline areas, which is similar to hooded rats. Our results suggest that differences in the melanocyte presence among the skin regions should be considered when the results of gene expression analyses of albino rat skin are interpreted.

## Introduction

The coloration patterns observed in most terrestrial mammals arise from the diverse color combinations within their fur, which covers most of their body surfaces. Hair is produced by the division of hair-matrix cells in the hair matrix of hair follicles, where melanocytes produce melanin pigment and provide it for hair [1–3]. The presence of melanocytes and their production of melanin in the hair matrix thus give hair or fur its color and determine the coloration pattern of mammals. In the synthesis of melanin by melanocytes, tyrosinase catalyzes tyrosine to dopaquinone. The subsequent actions of melanogenesis-related enzymes such as tyrosinase and dopachrome tautomerase (Dct) catalyze dopaquinone to melanin. These enzymes are used to detect melanocytes, Dct in particular is well known as a specific marker for the detection of melanocyte lineages [4,5]. Tyrosinase is the first enzyme in the melanin synthesis pathway converting tyrosine to DOPA and to dopaquinone [6] and is thus considered the key enzyme in the pathway.

Albino rats are frequently used as laboratory animals. Their characteristic white coat and body are due to a mutation in the tyrosinase gene, which eliminates the tyrosinase enzyme activity, thus making melanocytes unable to produce pigment [7,8]. With the exception of this genetic mutation and its associated loss of enzymatic activity, albino rats are generally considered to have no obvious differences compared to wild-type pigmented rats. We speculated that albino melanocytes with no pigment production are present in the area in which pigment is produced, i.e., the hair matrix. However, our search of the relevant literature identified no thorough investigation of the presence of melanocytes in the hair matrix of albino rat hair follicles.

In addition, the coloration of some rat strains (e.g., hooded rats) shows patterns. For example, black and white coloration patterns cause the combination of black hairs containing melanocyte-produced melanin and white hairs that do not contain this melanin. We hypothesized that since albino rats have the tyrosinase gene without mutation, they should show colored hairs and coloration patterns such as uniform or mottled coloration because differentiated melanocytes must be at the hair matrix and ready to produce melanin if it were not for non-functioning tyrosinase. Kuramoto et al. reported most strains of albino rats are genetically from hooded rats [8], but it has not been known whether albino rats have a coloration pattern phenotype like hooded rats do; that is to say, it is not yet clear whether albino rats have a distribution pattern of hair follicles that contain melanocytes in their hair matrix area(s). We conducted the present study to (*i*) clarify whether melanocytes are in the hair matrix of albino rat hair follicles, and (*ii*) identify the distribution of melanocyte-containing follicles across all of the body skin in three strains of albino rats. The finding that melanocytes were present in albino rat skin, together with the differences in the distribution of melanocyte-containing follicles, provides useful information for the evaluation of melanocyte-related gene expression in albino rat skin.

## Materials and methods

### Animals and sample collection

**Fig 1:**
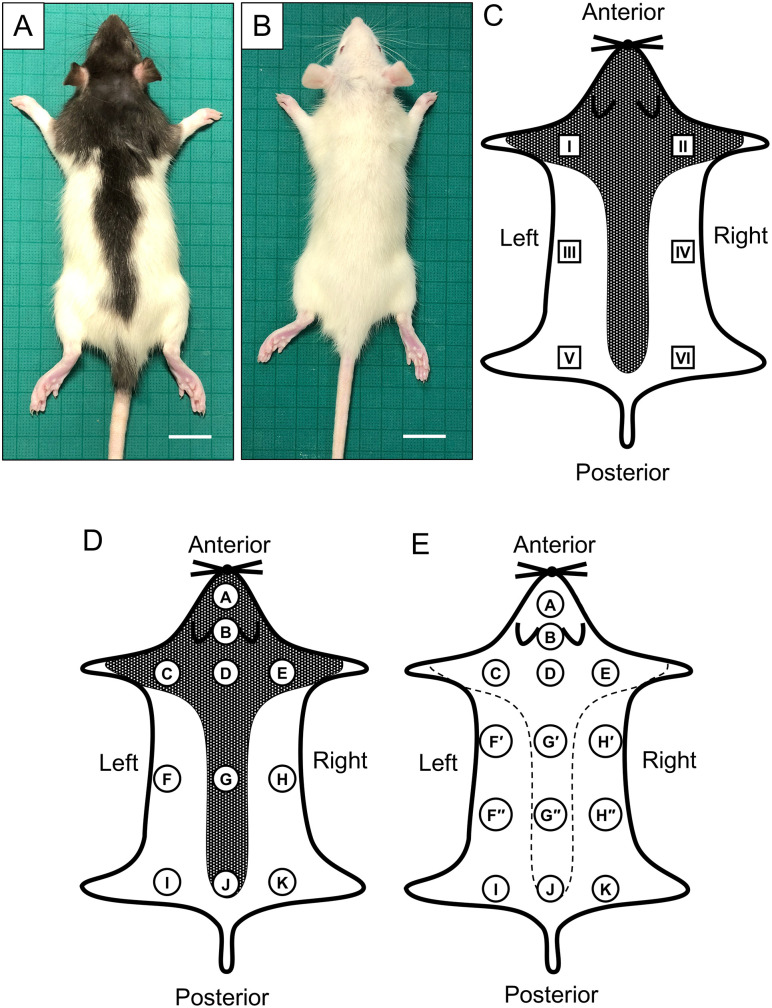
Sampled areas of skin. **A:** LE rats as a hooded rat strain. **B:** SD as an albino rat strain. **C–E:** Schematic diagrams of the rat dorsal view after skin samples were spread. The *black-gray area* in panels C and D: the region of the black hair color of LE rats. The *dotted line* in panel E: the estimated boundary in the albino strains SD, Wistar, and F344. The skin areas indicated by *squares and circles* were sampled for the RT-qPCR analysis and fluorescence immunohistochemistry, respectively. Areas F, G, and H were each divided into two separate sub-areas indicated by capital letters, with ‘ and “ used for the evaluation of the expression of the marker proteins. Scale bars: 2 cm.

All protocols for this study were approved by the ethics committee for vertebrate experiments at Azabu University (ID# 220308). We used four 5-week-old male rats of each of the following strains: outbred rat strains, Long-Evans (LE) rats (Iar:Long-Evans, Institute for Animal Reproduction, Kasumigaura, Ibaraki, Japan; Fig 1A), Sprague Dawley (SD) rats (Slc:SD, Japan SLC, Hamamatsu, Japan; Fig 1B) and Wistar rats (Slc:Wistar, SLC), an inbred strain, F344 (F344/NSlc, SLC). They were deeply anesthetized with a cocktail of medetomidine (0.3 mg/kg BW, Domitor, Nippon Zenyaku Kogyo, Fukushima, Japan), midazolam (4.0 mg/kg BW, Fuji Pharma, Toyama, Japan) and butorphanol (5.0 mg/kg BW, Vetrphale, Meiji Animal Health Co., Tokyo, Japan) administrated via intraperitoneal injection, and euthanized by cervical dislocation.

Skin pieces cut into 1 cm x 0.5 cm squares from six body areas (Fig 1C and D) were taken from euthanized rats and stored in a deep freezer at −80°C until the total RNA extraction for the reverse transcription quantitative PCR analysis. Eleven and 14 areas of skins were removed from LE and albino strain rats, respectively, as indicated in Fig 1C and D. The skin samples were immersed in 4% paraformaldehyde in 0.1 M phosphate buffer (pH 7.4) overnight at 4°C. They were then rinsed with phosphate-buffered saline (PBS, pH 7.4) and subjected to paraffin sectioning.

### RT-qPCR analysis

For the reverse transcription-quantitative polymerase chain reaction (RT-qPCR) analysis, total RNA was extracted from the rat skin samples with Sepasol-RNA I Super G (cat. #09379−97, Nacali Tesque, Kyoto, Japan) according to the manufacturer’s protocol. The total RNA was diluted to 250 µg/mL. A premixture was adjusted with 2 µL of total RNA, 1 µL of Oligo(dT)primer (cat. #SO132, Thermo Fisher Scientific, Tokyo), 4 µL of 2.5 mM dNTP Mix (cat. #4030, Takara Bio, Shiga, Japan), and 6 µL of diethyl pyrocarbonate (DEPC) water. After incubation at 65°C for 5 min and cooling on ice, the reagents of the SuperScript III Reverse transcriptase kit (cat. #18080093, Thermo Fisher Scientific), 4 µL of 5x First-Strand Buffer, 2 µL of 0.1 M DTT, and 1 µL of SuperScript III RT were added into the tube. The mixture was incubated at 50°C for 60 min and then at 70°C for 15 min to obtain cDNA.

A qPCR analysis was then performed to detect the expression amounts of Dct, tyrosinase, and glyceraldehyde-3-phosphatase dehydrogenase (GAPDH) in the mixture with SYBR Premix Ex Taq II (Takara Bio) using the following primer pairs. Dct forward primer: CAGATTGCCAATTGCAGCGT, Dct reverse primer: CGTTGCCAATGAGTCTCTGGA. Tyrosinase forward primer: TGGCACAGACTGTTCTTGCT, tyrosinase reverse primer: GACGCTGGGCTGAGTAAGTT. GAPDH forward primer: ACCACCAACTGCTTAGCC, GAPDH reverse primer: ATCACGCCACAGCTTTCC. The reaction cycle was 95°C for 30 sec as the initial denaturation, 95°C for 5 sec as a denaturation step, and 60°C for 34 sec as the annealing step. Each expression level was standardized with the expression amount of GAPDH. The results are presented as the expression levels in the skin samples from each body area as the relative value against that in area VI.

### Fluorescence immunohistochemistry

For the fluorescence immunohistochemical analysis, fixed skin samples were embedded in paraffin and then sectioned at 5 µm to obtain three paraffin sections every 1 mm in each area. After deparaffinization, the sections were processed for fluorescence immunohistology as described [9]. The epitope retrieval of sections was performed in 10 mM citrate buffer (pH 6.0) for 15 min at 95°C, and sections were incubated in blocking buffer for 1 hr at room temperature to diminish unspecific reaction. Rabbit monoclonal anti-Dct antibody (1: 2000, cat. #ab221144, clone#EPP21986, Abcam, Cambridge, UK) or rabbit polyclonal anti-Ki67 antibody (1:200, cat#ab66155, Abcam) reacted as a primary antibody in a moisture chamber overnight at 4°C. After a rinse with PBS and 70% ethanol, the sections were stained with 0.1% Sudan Black B (cat. #192−04412, Fujifilm-Wako Pure Chemicals, Osaka, Japan) in 70% ethanol for 5 min to reduce the auto-fluorescence of the sections and to visualize the histological structures of the hair follicles and skin in bright-field microscopy. The sections were then rinsed with PBS and incubated with Alexa 488-conjugated donkey anti-rabbit IgG antibody (1:200, Jackson ImmunoResearch Lab, Philadelphia, PA, USA) and 4’,6-diamidino-2-phenylindole (DAPI, cat. #D9542, Sigma Aldrich, St. Louis, MO). Other sections were processed without primary antibody reaction in parallel with the secondary antibody as a negative control. No specific reaction signals were observed on the negative control sections.

### Morphometry

In three sections from each rat body area, the total number of hair follicles containing a hair papilla was counted under a microscope. Hair follicles that contained both a hair papilla and anti-Dct antibody-positive cells (melanocytes) were also counted, and for the measurement of the proportion of these follicles, this number was divided by the total number of hair follicles with a hair papilla.

### Statistical analyses

The expression level of mRNA and the population of the hair follicles are presented as relative values, the mean ± standard deviation (SD)% (n = 4). After we conducted an analysis of variance (ANOVA), we analyzed the data by Tukey’s method to detect significant differences between the areas, p < 0.05.

## Results

### RT-qPCR analysis

**Fig 2:**
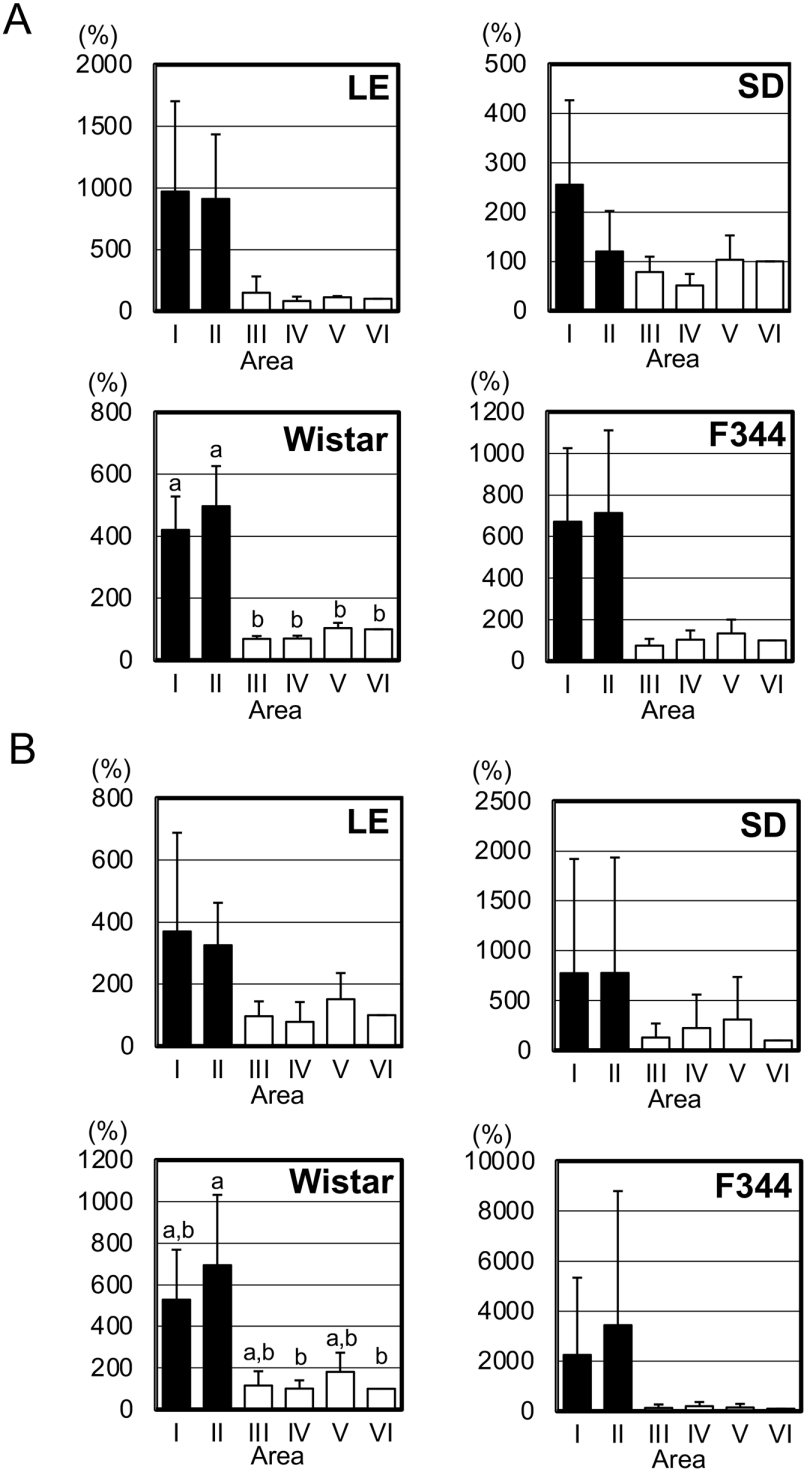
The Dct and tyrosinase mRNA expression levels in the rat skin areas. The expression levels of the melanocyte markers Dct **(A)** and tyrosinase **(B)** in the areas shown in Fig 1 were analyzed with by RT-qPCR. Data are mean ± SD (n = 4), relative to area VI. *x-axis:* the sampled skin areas. *y-axis:* the percentage of relative expression values. *Filled columns:* data from the black-hair area of LE rats or the estimated black-hair area of the albino strains (SD, Wistar and F344). *Unfilled columns:* data from the white-hair area of LE rats or the estimated white-hair area of the albino strains. *Differing letters:* p < 0.05 between groups.

In the skin samples from body areas I–VI of all four rat strains, i.e., Long-Evans (LE), Sprague Dawley (SD), Wistar, and F344 (Fig 1) we analyzed the mRNA expression levels of the melanogenesis-related enzymes Dct (S1 Table) and tyrosinase (S2 Table) to determine whether melanocytes are present in albino rats. The expression levels of Dct in the skin of the LE rats are illustrated in Fig 2A, and those of tyrosinase are depicted in Fig 2B. The expression levels of Dct and tyrosinase were higher in areas I and II than in the four other areas, but not significantly so.

In the SD rats’ skin samples, the expression levels of both Dct (Fig 2A) and tyrosinase (Fig 2B) in areas I and II were also higher compared to the other areas, but no significant difference was observed.

In the skin samples from the Wistar rats, the Dct expression levels (Fig 2A) and tyrosinase expression levels (Fig 2B) are also listed in S1 and S2 Tables. The Dct expression levels in areas I and II were significantly higher compared to those in the other areas, and the tyrosinase expression levels in areas I and II were also higher than those in the other areas, there was significant difference in areas II compared to areas IV or VI.

In the F344 rats’ skin samples, higher levels of both Dct (Fig 2A) and tyrosine (Fig 2B) gene expressions were observed in areas I and II compared to the other areas, but the differences were not significant (S1 and S2 Tables).

These RT-qPCR results indicate that melanocytes are present even in the skin of albino rats, and that their distribution may differ among areas.

### Bright-field microscopy and immunohistochemistry

**Fig 3:**
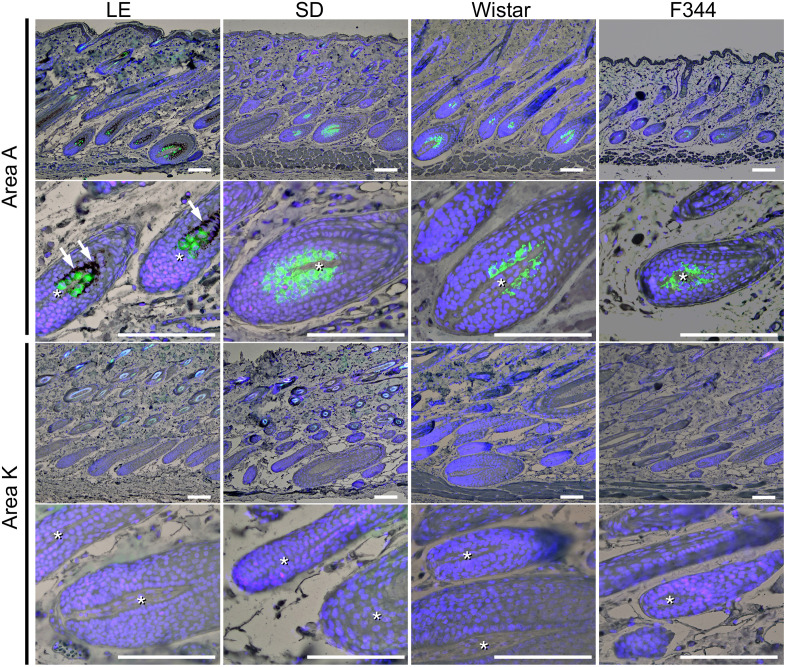
Fluorescence immunohistochemistry of rat skin to detect anti-Dct antibody-positive cells. The microscopic images were obtained by combining bright-field and dark-field microphotographs of skin sections double-stained with Sudan Black B and fluorescent immunohistochemistry with anti-Dct antibody (*green*) and DAPI (*blue*). Area A: a sampled skin area containing black hairs from LE rats or the estimated black hairs in the albino strains (SD, Wistar, and F344). Area K does not contain such hair. *Asterisks*: hair papillae in hair follicles. *Arrows:* melanin in hair follicles. All scale bars: 100 µm.

Skin sections from each rat strain were double-stained with Sudan Black B and subjected to a fluorescence immunohistochemistry evaluation for the observation of the general morphology of hair follicles to confirm the hair-cycle phase and for the detection of Dct expression (Fig 3). The results for area A and area K (Fig 3) are examples of the typical observation results for the black-hair (or putative black-hair) areas and white-hair (or putative white-hair) areas.

The examination using bright-field microscopy revealed hair follicles in all of the skin samples from the LE rats, and the samples from the three albino strains showed distinct hair bulbs and hair papillae (Fig 3, asterisks), with the hair bulbs clearly located in the subcutaneous tissue, suggesting that the hair follicles of these rats were in the anagen III–VI, during which melanocytes are active in melanin production. We observed melanin pigment in the hairs and in/around the hair matrix in black-hair areas, i.e., areas A, B, C, D, E, G, and J of the skin from LE rats, and not in putative black-hair areas, i.e., areas A, B, C, D, E, G’, G“ and J of the skin from all four rat strains. Melanin was not also observed in white-hair areas, i.e., areas F, H, I, and K of the LE rats and the putative white-hair areas F’, H’, F, H, I, and K of the albino rat strains.

In the LE rats, cells that were immunopositive for Dct were necessarily present in the hair matrix of melanin-containing hair follicles (Fig 3, area A), whereas they were absent from the hair matrix of the white follicles. In the three strains of albino rats, cells that were immunopositive for Dct were easily observed in the hair matrix of follicles in areas A–E, G’, G“, and J (Fig 3). In contrast, in the hair matrix within the areas presumed to contained white-hair follicles, i.e., areas F’, F”, H’, H”, I, and K in the albino strains, we observed hardly any cells immunopositive for Dct.

These immunohistochemistry results show that melanocytes are present even in the skin of albino rats and support a possible difference in their distribution among areas.

### Morphometry

**Fig 4:**
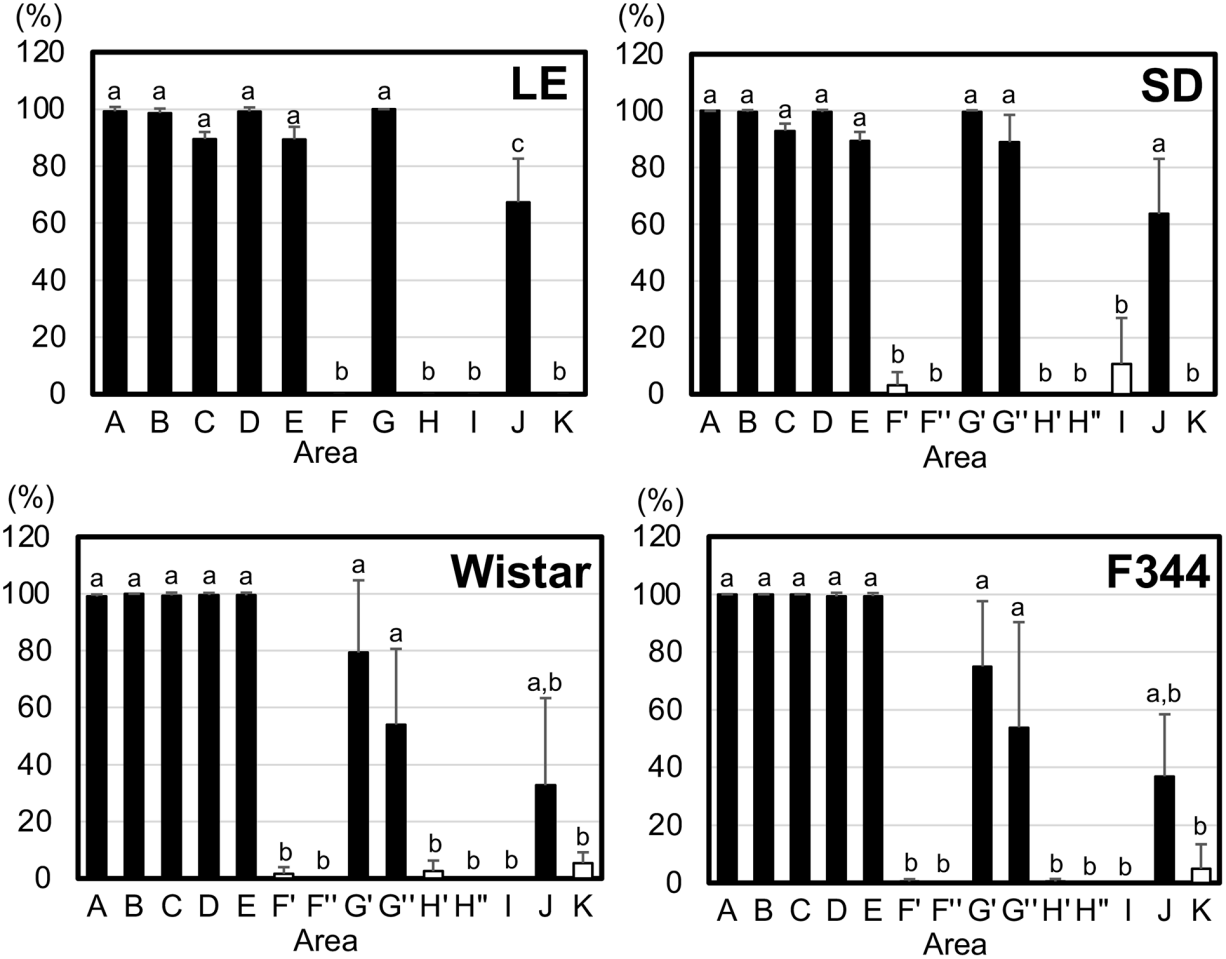
The population of hair follicles containing anti-Dct antibody-positive cells. Data are the population of hair follicles containing the positive cells relative to the total number of hair follicles, expressed as the mean ± SD (n = 4). The x- and y-axes indicate the sampled skin areas and the percentage of hair follicles containing positive cells, respectively, as described in the Materials and Methods section. *Black columns:* the data from black-hair areas of LE rats or the estimated black-hair areas of the albino strains SD, Wistar, and F344. *Unfilled columns:* data from the white-hair area of LE rats or the estimated white-hair area of the albino strains. *Differing letters:* p < 0.05 between groups.

Given the observed differences in the mRNA expression levels of Dct and tyrosinase across different body areas of the rats and the tendency for the distribution of Dct-immunopositive cells to vary regionally, we next quantified the numbers of hair follicles. The total follicle number was defined as the number of hair follicles exhibiting obvious hair papillae. Within these follicles, the number of hair follicles containing Dct-immunopositive cells was also counted, and the ratio of hair follicles containing positive cells to the total follicle number was calculated (Fig 4). The numbers of sampling areas for the immunohistochemistry and the detailed morphometric analysis were increased to 11 areas in the LE rats (Fig 1D) and 14 areas in the three albino strains (Fig 1E) compared to the number of areas used for the RT-qPCR sampling (Fig 1C). The ratios of hair follicles containing Dct-immunopositive cells in the four rat strains are also presented in S3 Table. Across all strains, the ratio in area A was significantly higher than those in areas F’, F“, H’, H”, I, and K. In the areas located at the rats’ midline, the ratio tended to decrease gradually toward the caudal end. These morphometric results demonstrate significant differences in their distribution among areas.

In general, melanocytes are present with melanin production activity in the hair matrix during the anagen III–VI but are absent during the catagen and telogen phases [3,10–12]. Although the follicle hair cycle was evaluated as being in the anagen phase using simple bright-field microscopy, the follicles in areas with few or no Dct-immunopositive cells could actually be in phases other than anagen if there is no evidence of cell proliferation in the hair matrix. Therefore, to address the hair cycle in these areas, we investigated Ki67 expression, a well-established proliferation marker expressed in actively cycling cells, with fluorescence immunohistochemistry. Ki67-positive nuclei were consistently detected in the hair matrix cells of all hair bulbs observed in these areas (S2 Fig.). The bright-field microscopy showed the hair bulbs located in subcutaneous tissue (Fig 3). These results indicate that the hair follicles were in the anagen III–VI and lacked Dct-immunopositive cells in the hair matrix.

## Discussion

To the best of our knowledge, this study provides the first report of the presence of melanocytes in hair follicles and the distribution of hair follicles containing melanocytes in albino rats. In the three albino strains SD, Wistar, and F344, the mRNA expressions of the melanocyte markers Dct and tyrosinase were detected in skin samples by the RT-qPCR analysis, and Dct-immunopositive cells were revealed in the hair matrix by fluorescent immunohistochemistry. Generally, melanocytes express Dct in the hair matrix of anagen-phase hair follicles [3,10–12], and hooded rats have melanocytes in the hair matrix [13]. Melanocyte stem cells in hair follicle bulge divide and migrate into the hair matrix in synchrony with the hair cycle, and during the anagen phase they differentiate to melanocytes producing melanin pigment in the hair matrix. Experimental animals that are albino generally have a tyrosinase gene mutation and cannot produce melanin; their hair or fur is thus white. However, albino mice have melanocytes without producing melanin [14]. Although differentiated melanocytes do not produce melanin pigment, the presence of these cells has been demonstrated even in albino rats.

Immunohistochemistry using an antibody against PNL2, a melanocyte marker, detected immunopositive cells in the rat iris and choroid of the eyeball, eyelid, anus, scrotum, and muzzle rhinarium as well as cells showing weak immunoreactivity in the epidermal basal layer and hair bulbs of the whole body [15]. In our present investigation, the immunohistochemical analysis using a more widely used melanocyte marker, i.e., anti-Dct antibody, demonstrated the presence of cells showing clear immunoreactivity in the hair matrix of the rat hair follicles.

Melanocytes in the hair matrix actively produce melanin during the anagen phase [16], and the expression levels of Dct and tyrosinase are greatly increased during this phase, followed by a gradual decrease toward the telogen phase [11]. We observed that although whole follicles were histologically in the anagen phase, the mRNA expression levels of Dct and tyrosinase showed high variability, and no significant difference in these levels were identified among the body areas. This variation in expression levels may be explained by differences in the activities of melanocytes across gradual sub-phases within the anagen phase. However, our detection of the melanocyte markers’ expressions supports the existence of melanocytes in the skin of the albino rats. Taken together, our findings demonstrate the presence of melanocytes with no melanin production in the hair matrix of three strains of albino rats. Melanin synthesis in these cells could be restored following the introduction and expression of the wild-type tyrosinase gene, for instance, by transgenesis or knock-in.

In the LE rats examined herein, anti-Dct immunopositive cells, i.e., melanocytes, were present in the hair matrix of black-hair areas A–E, G, and J, and not in the hair matrix of the white-hair areas F, H, I, or K. The ratio of hair follicles containing melanocytes was higher in putative black-hair areas (areas A–E, G’, G“ and J) than in putative white-hair areas (F’, H’, F”, H”, I, and K), even in the three albino strains. The ratio of hair follicles containing melanocytes in midline areas of the LE rats and the albino rats gradually decreased toward the caudal end. The black-hair area is on the rat’s midline and gradually narrows toward the tail. Moreover, the end of the black-hair area (e.g., around area J) contained a white-hair area and looked discontinuous (Fig 1A) [17]. This LE rat midline composition agrees with the gradual alteration of the LE rats’ ratio of hair follicles containing Dct-immunopositive cells and even with that of the albino rats’ ratios, as depicted in Fig 4.

In addition, some LE rats have a black-hair area from the midline to the lateral side of the femur [17], like areas I and K in the present study (S1 Fig). The morphological analysis that we performed detected Dct-immunopositive cells in area I of the SD rats and in area K of the Wistar rats and F344 rats, which may indicate that SD, Wistar, and F344 rats exhibit a variety of black-hair profiles in areas I and K. This variety among the albino rat strains is highly similar to that observed in LE rats.

**Fig 5:**
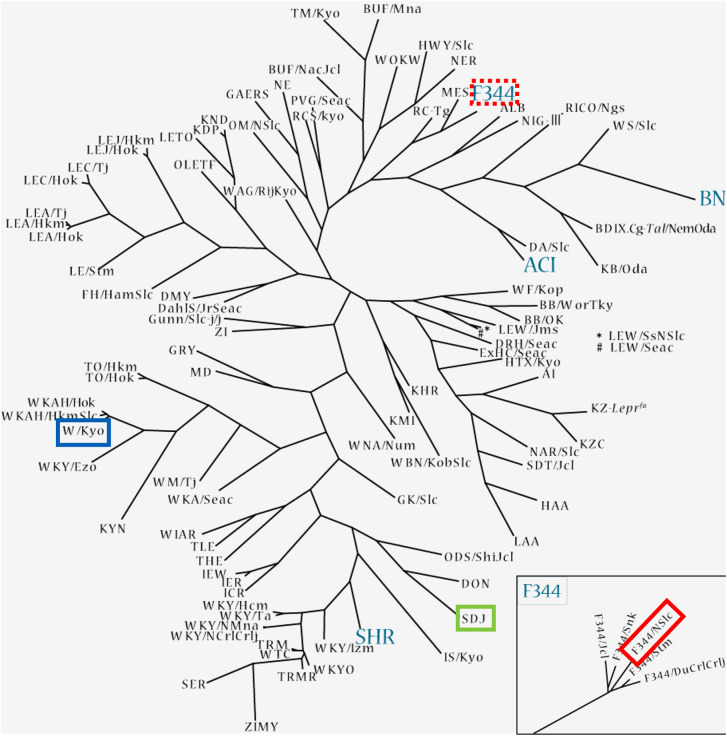
The rat linage tree of 132 rat inbred strains in Japan’s National BioResource Project. This tree is adapted from the National BioResource Project (NBRP) website, with permission (see Note below). This tree was constructed based on genome profiles. The strains genetically close to Slc:SD, Slc:Wistar, and F344/NSlc are enclosed in *green*, *blue*, and *red boxes*, respectively. *Right lower panel:* the detail strains in F344, *bordered with a red dotted line*. Note: Adapted with permission from the NBRP website [18].

Kurotaki et al. detected melanocytes in the epidermal basal layer and the hair bulbs throughout the rat body [15]. In the present study, we observed a high amount of hair bulbs containing Dct-immunopositive cells in the head, dorsal thorax, and dorsal midline areas of the albino rats. The 117 known strains of albino rats all carry the hooded allele, which contributes to the hooded phenotype and suggests that the albino rats are derived from hooded rats [8]. The albino strains, SD, Wistar and F344, used in the present study are partially present in the rat lineage tree (Fig 5) [18]. The three albino strains used herein were obtained from SLC, Inc., an experimental animal supplier in Japan. The Slc:SD strain has been maintained since its introduction from Charles River Laboratories in 1968 and is close to the strain SDJ/Hok (Fig 5, green square) in the rat lineage tree. The Slc:Wistar strain was introduced by the Institute of Medical Science, The University of Tokyo in 1968, and is close to the W/Kyo (Fig 5, blue square). The F344/NSlc strain is the same strain as that in the rat lineage tree (Fig 5, red square). These closely related strains show a large average genetic distance in the tree. Taken together, our results suggest that all strains that are suggested to be genetically derived from hooded rats should show the same distribution of hair bulbs containing non-melanin-producing melanocytes. However, not all of the albino rat strains used in animal experiments were examined for the distribution patterns of melanocyte-containing hair follicles in the present study. The distribution of hair-follicle melanocytes throughout the body described in the report by Kurotaki et al. [15] may be explained as follows; the strain that they used has a phenotype that differs from those of the strains used in our present investigation. Therefore, the patterns in other strains should be addressed in future work.

In human, there are eight recognized subtypes of albinism, causing by mutations in different genes. The albinism type of the albino rat strains, 117 strains, is oculocutaneous albinism (OCA) type 1, lack of melanin production throughout life by Tyr gene mutation [8]. The melanocyte distribution pattern in albino rats, resembling the hooded phenotype and shown in the present study, is generated by the hooded allele, which causes dysregulation of Kit expression, as indicated by Kuramoto et al. [8]. Care should be taken in interpreting the results derived from employing albino rats as a straightforward model of human OCA1.

The findings of this study strongly support the possibility that albino rat strains originated from hooded rats. The data that we obtained regarding melanocyte-containing follicle areas at the rat head, dorsal thorax and dorsal midline will contribute to future dermatological research using albino rats, since results concerning the skin can be affected by the presence of melanocytes and their gene expression, thus requiring careful interpretation. Additionally, since only male rats were investigated in the present study, the presence of melanocytes and the distribution of melanocyte-containing hair follicles in female rats remain unclear. The expression of the melanocyte marker and localization of the positive signals demonstrated the presence of melanocytes in the hair matrix of the black-hair areas of LE rats and the putative black-hair areas of albino rats; however, it remains unclear whether these melanocytes in albino rats are fully active in melanogenesis and whether the tyrosinase gene mutation in these rats is accompanied by other factors affecting melanogenesis. Further studies using various albino strains, including both sexes and transgenic approaches introducing a normal tyrosinase gene, will be necessary to address these issues.

## Supporting information

S1 Fig External pattern appearance of LE rats used in the present study. Scale bar: 2 cm.
(PDF)

S2 Fig Fluorescence immunohistochemistry of rat skin to detect anti-Ki67 antibody-positive cells. The microscopic images were obtained by combining bright-field and dark-field microphotographs of skin sections double-stained with Sudan Black B and fluorescence immunohistochemistry with anti-Ki67 antibody (*green*) (A to Z). The panels of the lowercase letters indicate magnified images of the boxed areas in the uppercase panels, showing immunofluorescence staining under dark-field microscopy in the hair bulbs (anti-Ki67 antibody positive, *green*; DAPI, *blue*). Arrows indicate the regions containing melanin pigment and asterisks denote the dermal papilla. Nuclei within the hair matrix exhibited positive signal for the anti-Ki67 antibody. All scale bars: 100 µm.
(PDF)

S1 Table The levels of Dct expression in skin samples from the six body areas of three albino rat strains (SD, Wistar, and F344) and the non-albino strain LE. (DOCX)

S2 Table The levels of tyrosinase expression in skin samples from the six body areas of three albino rat strains (SD, Wistar, and F344) and the non-albino strain LE. (DOCX)

S3 Table The ratios of hair follicles containing Dct-immunopositive cells. (DOCX)

## References

[1] Hosaka C, Kunisada M, Koyanagi-Aoi M, Masaki T, Takemori C, Taniguchi-Ikeda M, et al. Induced pluripotent stem cell-derived melanocyte precursor cells undergoing differentiation into melanocytes. Pigment Cell Melanoma Res. 2019;32(5):623–33. doi: 10.1111/pcmr.12779 30843370

[2] Slominski A, Wortsman J, Plonka PM, Schallreuter KU, Paus R, Tobin DJ. Hair follicle pigmentation. J Invest Dermatol. 2005;124(1):13–21. doi: 10.1111/j.0022-202X.2004.23528.x 15654948 PMC1201498

[3] Tobin DJ, Slominski A, Botchkarev V, Paus R. The fate of hair follicle melanocytes during the hair growth cycle. J Investig Dermatol Symp Proc. 1999;4(3):323–32. doi: 10.1038/sj.jidsp.5640239 10674391

[4] Nishikawa-Torikai S, Osawa M, Nishikawa S-I. Functional characterization of melanocyte stem cells in hair follicles. J Invest Dermatol. 2011;131(12):2358–67. doi: 10.1038/jid.2011.195 21753783

[5] Steel KP, Davidson DR, Jackson IJ. TRP-2/DT, a new early melanoblast marker, shows that steel growth factor (c-kit ligand) is a survival factor. Development. 1992;115(4):1111–9. doi: 10.1242/dev.115.4.1111 1280558

[6] Cooksey CJ, Garratt PJ, Land EJ, Pavel S, Ramsden CA, Riley PA, et al. Evidence of the indirect formation of the catecholic intermediate substrate responsible for the autoactivation kinetics of tyrosinase. J Biol Chem. 1997;272(42):26226–35. doi: 10.1074/jbc.272.42.26226 9334191

[7] Blaszczyk WM, Arning L, Hoffmann K-P, Epplen JT. A Tyrosinase missense mutation causes albinism in the Wistar rat. Pigment Cell Res. 2005;18(2):144–5. doi: 10.1111/j.1600-0749.2005.00227.x 15760344

[8] Kuramoto T, Nakanishi S, Ochiai M, Nakagama H, Voigt B, Serikawa T. Origins of albino and hooded rats: implications from molecular genetic analysis across modern laboratory rat strains. PLoS One. 2012;7(8):e43059. doi: 10.1371/journal.pone.0043059 22916206 PMC3420875

[9] Horio T, Ozawa A, Kamiie J, Sakaue M. Immunohistochemical analysis for acetylcholinesterase and choline acetyltransferase in mouse cerebral cortex after traumatic brain injury. J Vet Med Sci. 2020;82(6):827–35. doi: 10.1292/jvms.19-0551 32321871 PMC7324811

[10] Tobin DJ, Hagen E, Botchkarev VA, Paus R. Do hair bulb melanocytes undergo apoptosis during hair follicle regression (catagen)?. J Invest Dermatol. 1998;111(6):941–7. doi: 10.1046/j.1523-1747.1998.00417.x 9856800

[11] Botchkareva NV, Khlgatian M, Longley BJ, Botchkarev VA, Gilchrest BA. SCF/c-kit signaling is required for cyclic regeneration of the hair pigmentation unit. FASEB J. 2001;15(3):645–58. doi: 10.1096/fj.00-0368com 11259383

[12] Hirobe T. Structure and function of melanocytes: microscopic morphology and cell biology of mouse melanocytes in the epidermis and hair follicle. Histol Histopathol. 1995;10(1):223–37. 7756740

[13] Kostanecki W, Radwan I, Mroczkowski T. The effect of X-ray irradiation upon the epithelial melanin unit of the hair bulb in hooded rat. Arch Dermatol Res (1975). 1976;256(3):297–303. doi: 10.1007/BF00572496 984882

[14] Yamamoto H, Takeuchi S, Kudo T, Sato C, Takeuchi T. Melanin production in cultured albino melanocytes transfected with mouse tyrosinase cDNA. Jpn J Genet. 1989;64(2):121–35. doi: 10.1266/jjg.64.121 2517217

[15] Kurotaki T, Tomonari Y, Kanno T, Wako Y, Tsuchitani M. A novel immunohistochemical marker of normal and neoplastic melanocytes in formalin-fixed, paraffin-embedded tissues of albino rats. Vet Pathol. 2008;45(3):383–7. doi: 10.1354/vp.45-3-383 18487499

[16] Slominski A, Paus R, Plonka P, Chakraborty A, Maurer M, Pruski D, et al. Melanogenesis during the anagen-catagen-telogen transformation of the murine hair cycle. J Invest Dermatol. 1994;102(6):862–9. doi: 10.1111/1523-1747.ep12382606 8006449

[17] Wikramanayake TC, Amini S, Simon J, Mauro LM, Elgart G, Schachner LA, et al. A novel rat model for chemotherapy-induced alopecia. Clin Exp Dermatol. 2012;37(3):284–9. doi: 10.1111/j.1365-2230.2011.04239.x 22409523 PMC3305180

[18] The National Bio Resource Project for the Rat in Japan. The rat strain linage tree. Web site. https://www.anim.med.kyoto-u.ac.jp/nbr/phylo.aspx. 2018. Accessed 2025 May 6.

