# Supplementary figures and images for: Regional differences in the distribution of melanocyte-containing hair bulbs in the skin of male albino rats
Source: PLoS One. 2025 Nov 5;20(11):e0336110. doi: 10.1371/journal.pone.0336110 (PMC12588474; doi:10.1371/journal.pone.0336110)

S1 Figure

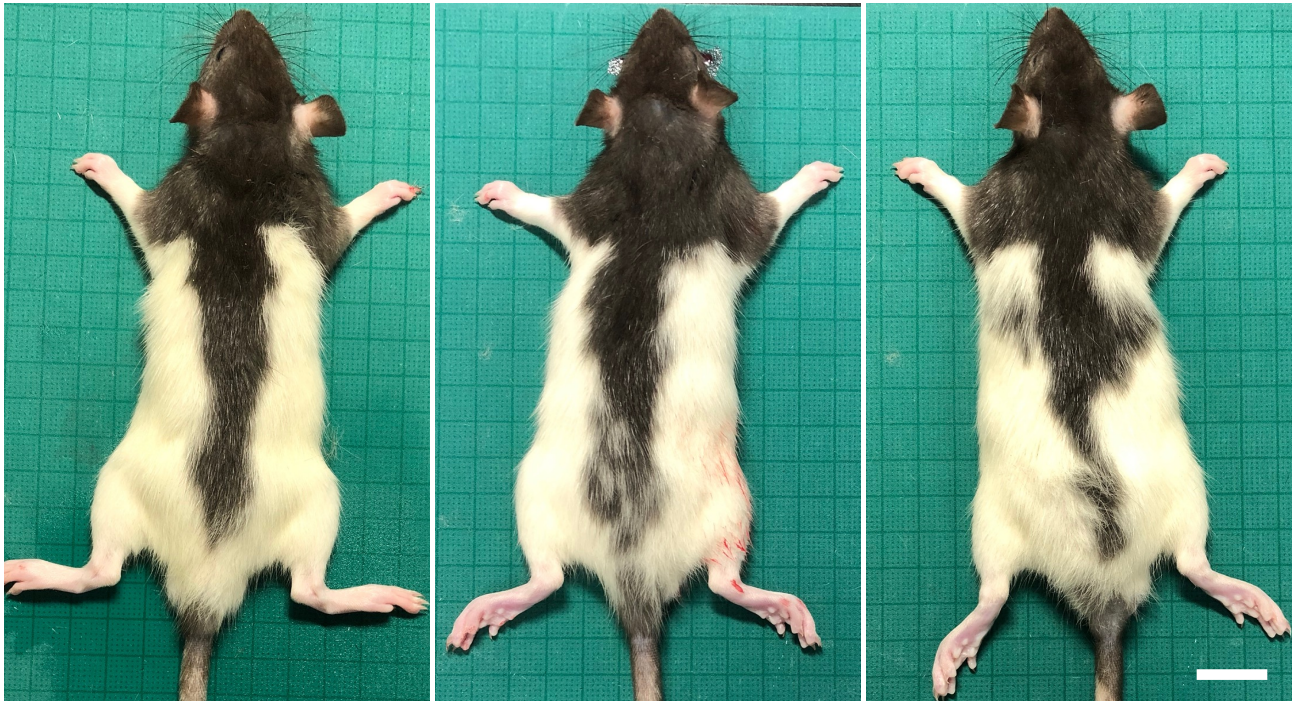

Supplement: S1 Fig — Scale bar: 2 cm. (PDF) [file pone.0336110.s001.pdf]
