# Supplementary material for: Regional differences in the distribution of melanocyte-containing hair bulbs in the skin of male albino rats
Source: PLoS One. 2025 Nov 5;20(11):e0336110. doi: 10.1371/journal.pone.0336110 (PMC12588474; doi:10.1371/journal.pone.0336110)

S2 Figure

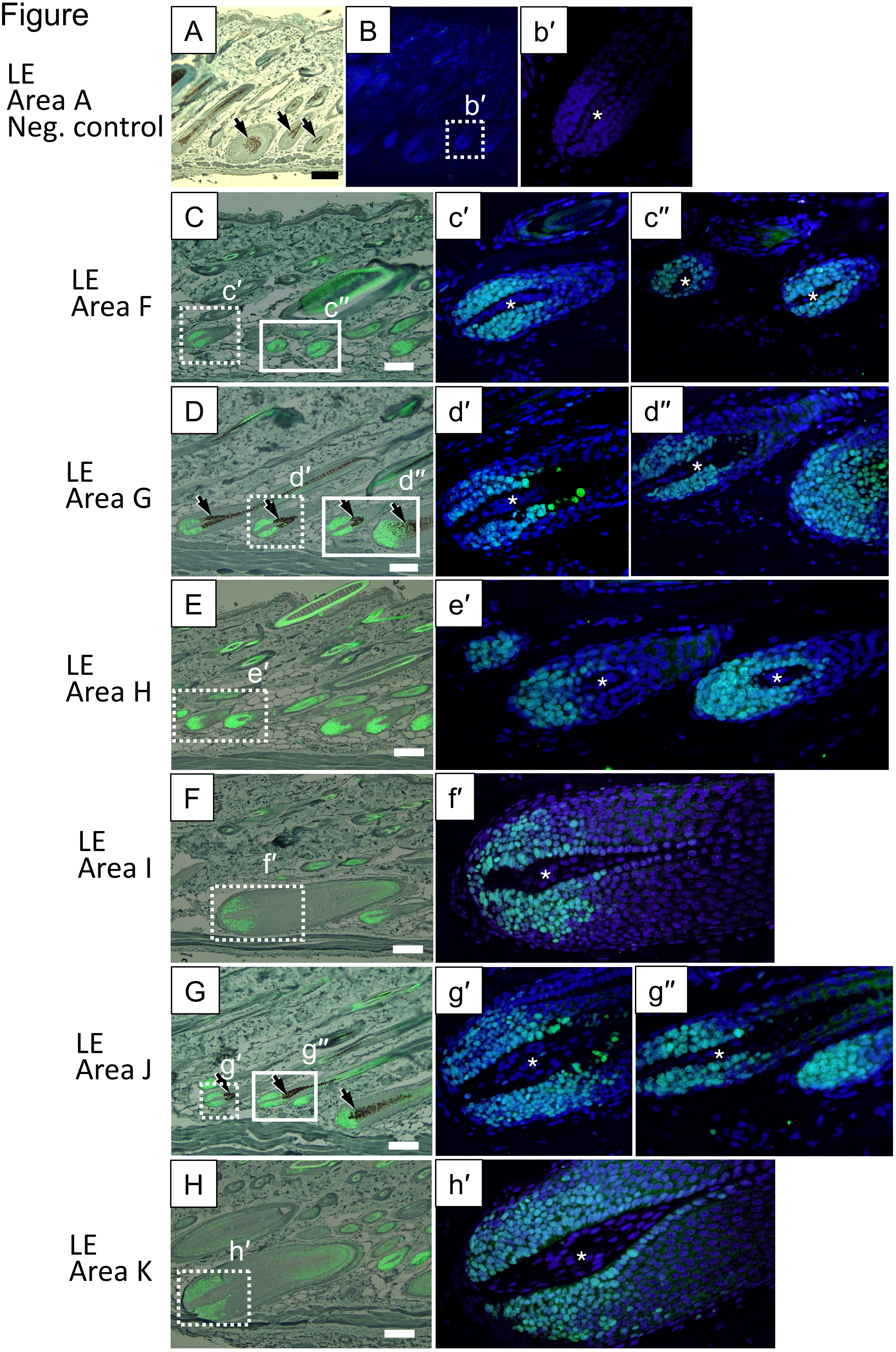

LE
Area A
Neg. control
A
B
b′
b′
LE
Area F
C
c′
c″
c′
c″
LE
Area G
D
d′
d″
d′
d″
LE
Area H
E
e′
e′
LE
Area I
F
f′
f′
LE
Area J
G
g′
g″
g′
g″
LE
Area K
H
h′
h′

S2 Figure (Continued)

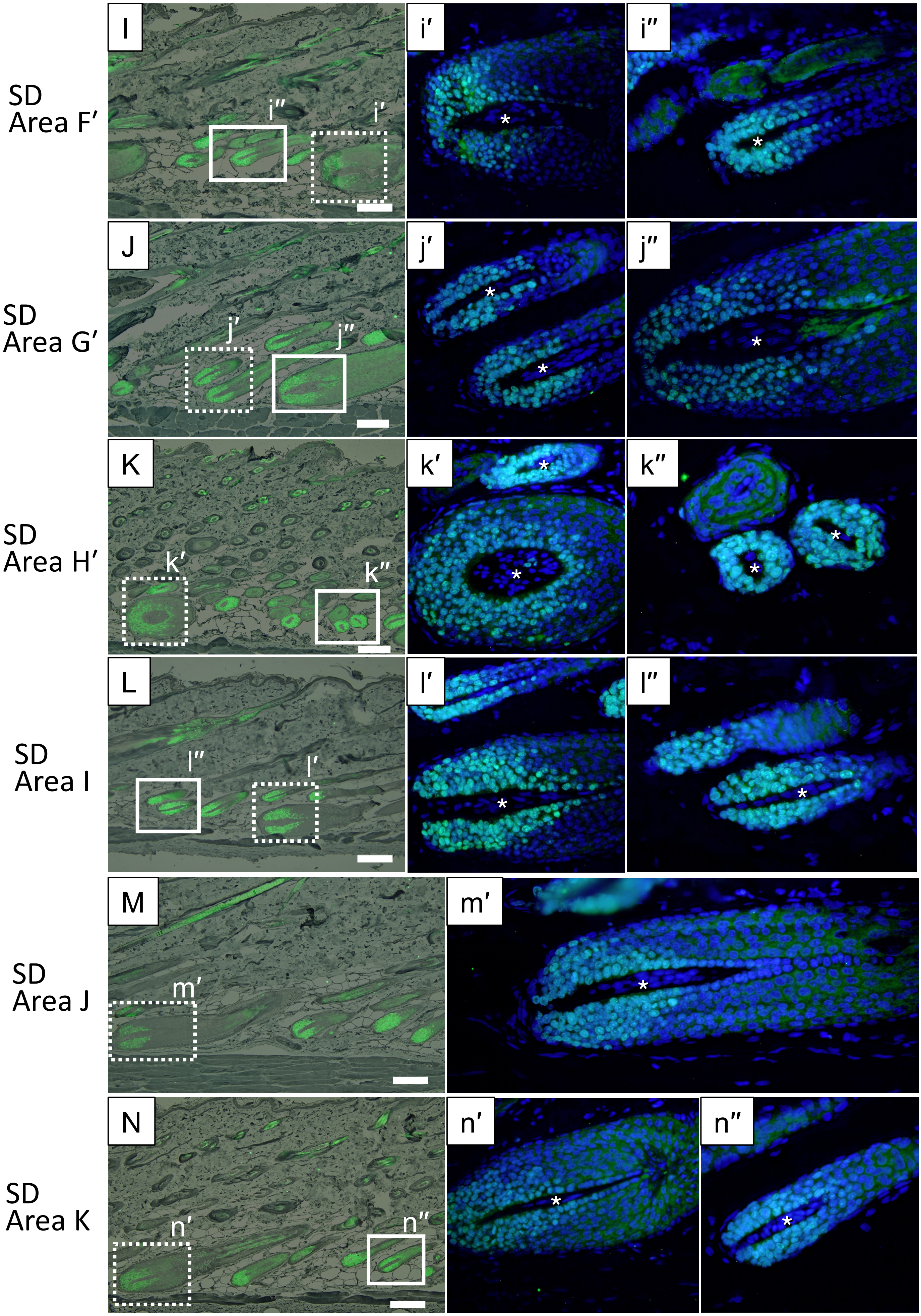

S2 Figure (Continued)

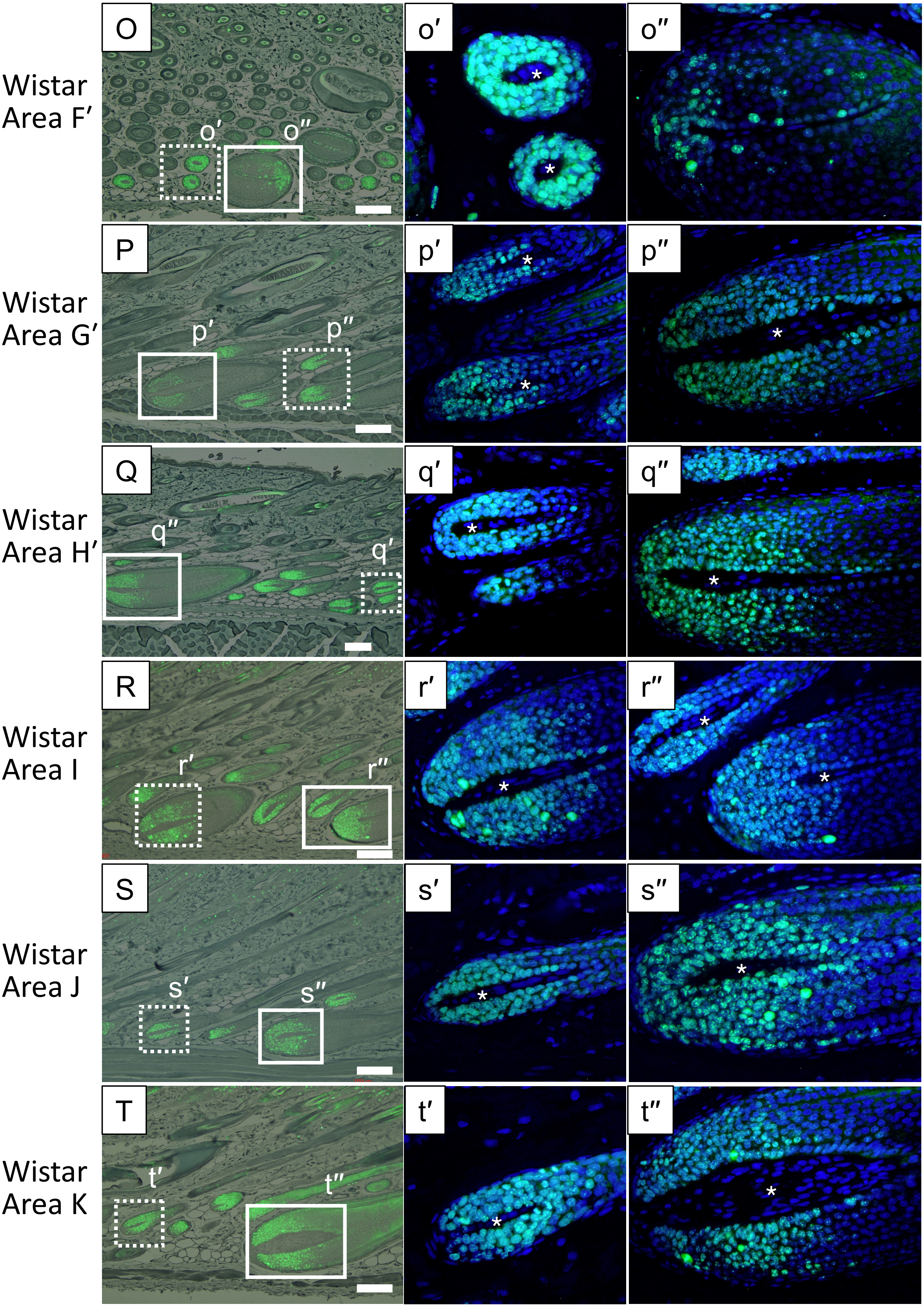

S2 Figure (Continued)

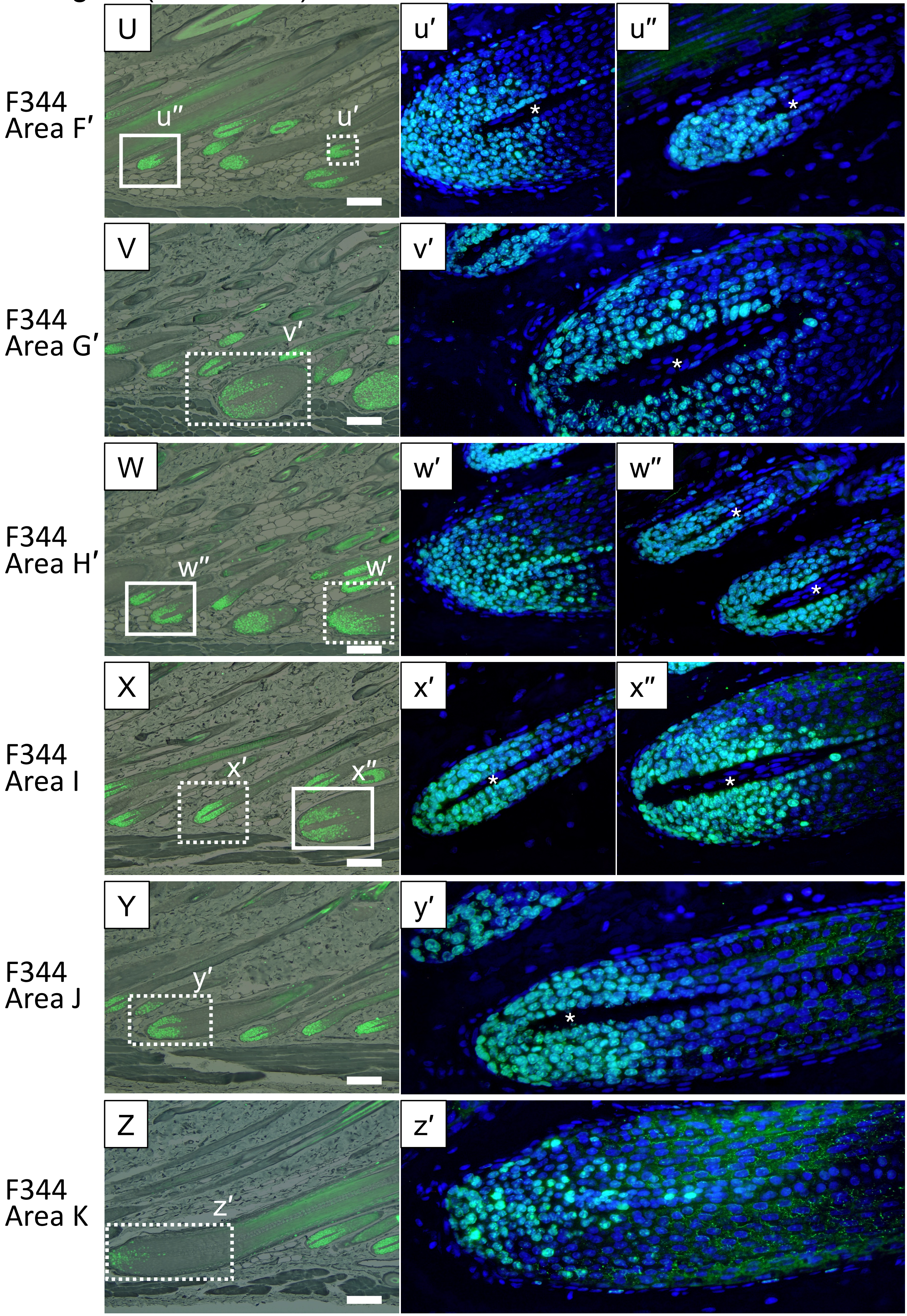

Supplement: S2 Fig — The microscopic images were obtained by combining bright-field and dark-field microphotographs of skin sections double-stained with Sudan Black B and fluorescence immunohistochemistry with anti-Ki67 antibody (green) (A to Z). The panels of the lowercase letters indicate magnified images of the boxed areas in the uppercase panels, showing immunofluorescence staining under dark-field microscopy in the hair bulbs (anti-Ki67 antibody positive, green; DAPI, blue). Arrows indicate the regions containing melanin pigment and asterisks denote the dermal papilla. Nuclei within the hair matrix exhibited positive signal for the anti-Ki67 antibody. All scale bars: 100 µm. (PDF) [file pone.0336110.s002.pdf]
