# Supplementary material for: Regional differences in the distribution of melanocyte-containing hair bulbs in the skin of male albino rats
Source: PLoS One. 2025 Nov 5;20(11):e0336110. doi: 10.1371/journal.pone.0336110 (PMC12588474; doi:10.1371/journal.pone.0336110)
Supplement: S1 Table — (DOCX) [file pone.0336110.s003.docx]

**S1 Table. The levels of Dct expression in skin samples from the six body areas of three albino rat strains (SD, Wistar, and F344) and the non-albino strain LE**

|  | **LE** | **SD** | **Wistar** | **F344** |
| --- | --- | --- | --- | --- |
| Area I | 970.4 ± 732.7 | 255.5 ± 171.4 | 420.6 ± 107.9 | 671.2 ± 354.1 |
| Area II | 910.4 ± 524.0 | 120.1 ± 82.2 | 497.2 ± 129.4 | 713.1 ± 397.9 |
| Area III | 149.2  ± 132.3 | 78.4 ± 31.2 | 69.0 ± 9.2 | 74.9 ± 32.4 |
| Area IV | 81.7 ± 35.5 | 51.1 ± 23.3 | 70.2 ± 8.9 | 103.3 ± 44.5 |
| Area V | 112.1 ± 9.9 | 103.3 ± 49.4 | 103.8 ± 16.9 | 133.3 ± 66.6 |
| Area VI | 100.0 ± 0.0 | 100.0 ± 0.0 | 100.0 ± 0.0 | 100.0 ± 0.0% |

The six areas are illustrated in Fig. 1C. The data are percentages, mean ± SD (n=4 for each rat strain). LE: Long-Evans, SD: Sprague-Dawley.
