# Supplementary material for: Regional differences in the distribution of melanocyte-containing hair bulbs in the skin of male albino rats
Source: PLoS One. 2025 Nov 5;20(11):e0336110. doi: 10.1371/journal.pone.0336110 (PMC12588474; doi:10.1371/journal.pone.0336110)
Supplement: S2 Table — (DOCX) [file pone.0336110.s004.docx]

**S2 Table. The levels of tyrosinase expression in skin samples from the six body areas of three albino rat strains (SD, Wistar, and F344) and the non-albino strain LE**

|  | **LE** | **SD** | **Wistar** | **F344** |
| --- | --- | --- | --- | --- |
| Area I | 369.8 ± 318.4 | 774.5 ± 1145.8 | 528.6 ± 240.2 | 2251.9 ± 3087.4 |
| Area II | 325.4 ± 137.0 | 777.1 ± 1157.6 | 694.3 ± 338.3 | 3439.9 ± 5361.8 |
| Area III | 96.5 ± 47.9 | 129.5 ± 140.7 | 115.7 ± 68.4 | 132.5 ± 137.7 |
| Area IV | 78.3 ± 63.9 | 224.3 ± 335.8 | 100.9 ± 39.6 | 201.7 ± 166.2 |
| Area V | 151.3 ± 84.4 | 310.0 ± 426.5 | 181.3 ± 92.2 | 149.0 ± 144.8 |
| Area VI | 100.0  ± 0.0 | 100.0 ± 0.0 | 100.0 ± 0.0 | 100.0 ± 0.0 |

The six areas are illustrated in Fig. 1C. The data are percentages, mean ± SD (n=4 for each rat strain). LE: Long-Evans, SD: Sprague-Dawley.
