# Supplementary material for: Regional differences in the distribution of melanocyte-containing hair bulbs in the skin of male albino rats
Source: PLoS One. 2025 Nov 5;20(11):e0336110. doi: 10.1371/journal.pone.0336110 (PMC12588474; doi:10.1371/journal.pone.0336110)
Supplement: S3 Table — (DOCX) [file pone.0336110.s005.docx]

**S3 Table. The ratios of hair follicles containing Dct-immunopositive cells**

|  | **LE** | | **SD** | **Wistar** | **F344** |
| --- | --- | --- | --- | --- | --- |
|  | Black | White |  |  |  |
| Area A | 99.2 ± 1.6 | − | 100 ± 0.0 | 99.0  ± 0.7 | 100 ± 0.0 |
| Area B | 98.6 ± 1.6 | − | 99.6 ± 0.6 | 100 ± 0.0 | 100 ± 0.0 |
| Area C | 89.5 ± 2.5 | − | 93.0 ± 2.5 | 99.4 ± 1.0 | 100 ± 0.0 |
| Area D | 99.2 ± 1.5 | − | 99.5 ± 0.8 | 99.6 ± 0.8 | 99.3 ± 1.3 |
| Area E | 89.4 ± 4.4 | − | 89.4 ± 3.2 | 99.5 ± 1.0 | 99.4 ± 1.0 |
| Area F | − | 0.0 ± 0.0 | − | − | − |
| Area F' | − | − | 3.1 ± 4.6 | 1.2 ± 2.1 | 0.5 ± 0.8 |
| Area F'' | − | − | 0.0 ± 0.0 | 0.0 ± 0.0 | 0.0 ± 0.0 |
| Area G | 100 ± 0.0 | − | − | − | − |
| Area G' | − | − | 99.7 ± 0.5 | 79.0 ± 22.0 | 75.0 ± 22.6 |
| Area G'' | − | − | 88.9 ±  9.7 | 65.3 ± 30.2 | 53.9 ± 36.5 |
| Area H | − | 0.0 ± 0.0 | − | − | − |
| Area H' | − | − | 0.0 ± 0.0 | 4.1 ± 4.1 | 0.5 ± 0.9 |
| Area H'' | − | − | 0.0 ± 0.0 | 0.0 ±  0.0 | 0.0 ± 0.0 |
| Area I | − | 0.0 ± 0.0 | 10.7 ± 16.2 | 0.0 ± 0.0 | 0.0 ± 0.0 |
| Area J | 67.3 ± 15.3 | − | 63.6 ± 19.5 | 44.6 ± 33.5 | 36.9 ± 21.5 |
| Area K | − | 0.0 ± 0.0 | 0.0 ± 0.0 | 7.0 ± 4.3 | 4.9 ± 8.5 |

The data are percentages, mean ± SD (n=4 for each rat strain). LE: Long-Evans, SD: Sprague-Dawley.
